# Effect of curing conditions on cement based self-compacting mortar produced with mortar waste aggregate

**DOI:** 10.1016/j.heliyon.2024.e36423

**Published:** 2024-08-17

**Authors:** Melek Akgül

**Affiliations:** Department of Civil Engineering, Munzur University, Tunceli, Turkey

**Keywords:** Self-compacting mortar, Mortar waste aggregate, Heat curing, Microstructure, Mechanical properties, Durability properties

## Abstract

Concrete and mortar wastes, which have a large volume and economic value among construction demolition wastes, are the most targeted demolition waste group to be recycled. An important area where construction demolition waste can be utilized is self-compacting mortar (SCM) systems. SCMs are innovative and economical systems designed to minimize the labor requirements that are difficult to meet in the production process. In this study, mortar waste aggregate (MWA) obtained by mechanical crushing and grinding was used in SCM elements by substituting different ratios (5-10-20-30-40 %) by mass to aggregate. In this way, it was aimed to evaluate both the sustainability of MWAs and the usability of MWAs in SCMs, which are considered as a new production technology. The fresh and hardened mortar tests performed in the study are presented comparatively. The physical (dry unit volume weight, porosity), durability (capillary water absorption) and mechanical properties (flexural tensile, compressive strength) of the hardened SCM elements are based on the determinations made at 3, 7 and 28 test days and according to different curing conditions (water curing, air curing and heat curing).

In addition, X-ray Diffractometer (XRD) analysis was performed on specimens obtained from 0 %, 10 %, 20 % and 40 % MWA substituted specimens after heat curing (after 7 days water curing) and 28 days water curing. In the light of the data obtained, it is reported that SCM production with 10 % MWA substitution is feasible in terms of sustainability and engineering properties evaluated in this study.

## 1 Introduction

The growth data of the construction sector is recorded with an increasing momentum every year. Especially from the 1970s to 2023, many researchers have conducted numerous studies on the economic power and growth of the construction sector in countries [[1], [2], [3]]. The construction of new buildings, as well as the demolition or maintenance of old buildings, entails numerous costs. Construction demolition waste accounts for a large proportion of the waste generated in Europe [4]. The World Green Building Council estimates that more than 50 % of global waste and 35 % of landfill space comes from the construction sector [5]. The processes involved in the storage and disposal of waste from construction demolitions have a huge potential impact on both environmental and economic parameters. For this reason, many countries are developing alternative systems for waste management. In many countries, especially Malaysia and China, there are studies and incentives regarding the economic power of construction demolition waste and its presence in the waste management process [6]. Furthermore, according to the EU Waste Strategy, construction demolition waste is considered as one of the priority waste management processes [7]. Therefore, sustainable processes in the disposal and recycling of construction demolition waste are of utmost importance.

In the construction industry, which is thought to be responsible for the generation of approximately 6 billion tons of CO_2_ emissions annually, cement and aggregate, which are filling materials, are an important expense item [8]. The supply of aggregates from natural sources is limited and the use of aggregates in the construction industry is heavily dependent on natural resources. The annual burden of aggregates on the sector is 2.7 billion tons in the EU and 900 million tons in the US [9].

In recent years, there have been many studies on the search for new resources [[10], [11], [12], [13], [14], [15], [16], [17], [18]]. It aims to limit the use of aggregate by especially by using construction demolition waste that can be substituted for aggregate [19].

To reduce the need for cement and aggregate, which is a particularly important component in the production of mortar, concrete and reinforced concrete elements, construction demolition waste is preferred as a substitute product and researchers continue to investigate its use and appropriate substitution rates [20]. Recycled mortar waste (rMW) has superior effects on some engineering properties compared to concrete waste (CW), especially in the new cement matrix in which it is used. Studies on the use of MW in mortar elements have been carried out more widely in recent years [21]. Construction demolition waste is used in concrete and its derivatives [22,23] as well as mortar and its derivatives [24] with different applications and substitution rates [25]. In addition, studies are underway to evaluate the substitution of recycled products as thin materials by activation methods (mechanical, chemical and nano activations) of different material chemical matrix or component to ensure sustainable levels [26]. However, the processing of these materials requires secondary cost items.

Compared to Portland cement, fine material has a positive effect on compressive strength [27,28]. Moreover, the crushing and grinding of the material obtained as waste from concrete and mortar elements during the recovery process reveals the presence of a fine product representing a significant amount (40–60 %), which is a secondary cement product resulting from the presence of unreacted cement grains [24]. SCM is a special type of concrete that sets under the influence of its own weight and has a specific viscosity level [[10], [11], [12],^14^,^16^,^18^,^29^]. SCM systems are allocated with a high proportion of cement, fine aggregates, and viscosity modifiers. Usually, viscosity modifying admixtures play an important role in preventing the W/B ratio from increasing unnecessarily [30]. The use of MW can lead to a decrease in the viscosity of SCM elements or an increase in the amount of water not included in the chemical process. However, keeping the W/B ratio constant in the design and varying the viscosity regulator ensures that SCM systems meet fresh state criteria [21]. Increasing the proportion of fine aggregate increases the compressive and flexural strength. It also has a reducing effect on permeability [31,32]. Fly ash substitution in SCMs has a significant impact on rheology and durability, blending with fly ash improves filling capacity [33]. Viscosity-enhancing plasticizers have an impact on compressive strength, porosity and slump flow [34].

According to the evaluation of literature and experimental studies, it is possible to utilize construction demolition waste in different proportions without the need for secondary pre-treatment by mechanical crushing and grinding only.

Following the earthquakes in the Kahramanmaras province of Turkey on February 6, 2023, large quantities of construction waste were generated in 11 earthquake-affected provinces [35]. Approximately 15 million tons of waste generated is construction waste [36]. The storage and disposal of this waste is a major economic and environmental challenge [37]. After major earthquakes and urban transformation processes, recycling of construction rubble waste is important. Methods such as heat treatment, mechanical treatment [38] and carbonation [39,40] are used in the use of construction demolition waste as aggregate. However, while these methods are an alternative [39,41], they are not applicable for the treatment of large volumes of post-earthquake wastes. Because processes such as heat treatment are not feasible and economical for large construction demolition wastes. Therefore, it is more environmentally friendly, economical and sustainable to use construction waste as aggregate only after crushing and grinding.

Most procedures in MWA recycling are complex. These processes require a lot of time, cost and energy [42]. Furthermore, some equipment and facilities are not widely available in most waste concrete processing plants. Therefore, it is important to fundamentally study the effect of untreated MWA without any pretreatment or thermal activation on mortar properties. This experimental study aims to substitute MWA with fine aggregate in SCM systems. In this way, both the labor requirement targeted in the design of SCM systems will be reduced and the usability of recycled MWA will be tested. In the SCM systems produced in the study, MWA was substituted for fine aggregate in proportions of 5, 10, 20, 30 and 40 % by mass. In addition to the design and fabrication of the SCM elements, the fresh state properties were determined according to EFNARC [43] criteria. Furthermore, the physical and mechanical properties of the hardened SCM elements were determined at test days 3, 7 and 28 and according to different curing conditions. In addition to data evaluating water cure, air cure and thermal cure, XRD was performed on test day 28 with specimens containing 0, 10, 20, 40 % MWA substitution.

## 2 Material and method

In this section, the materials used in the SCM produced within the scope of the experimental study and the experimental methods applied are given in detail with subheadings.

### 2.1 Material

**Fig. 1:**
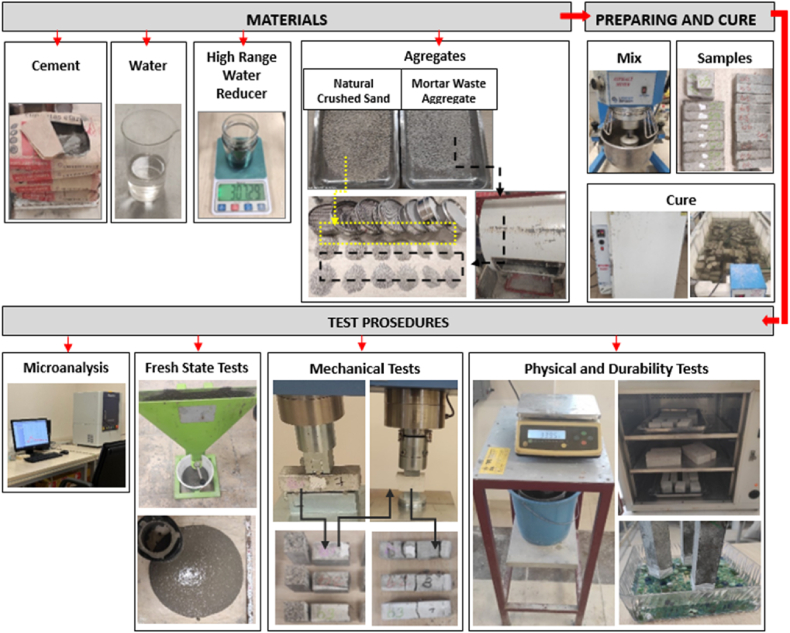
Workflow of the study.

**Fig. 2:**
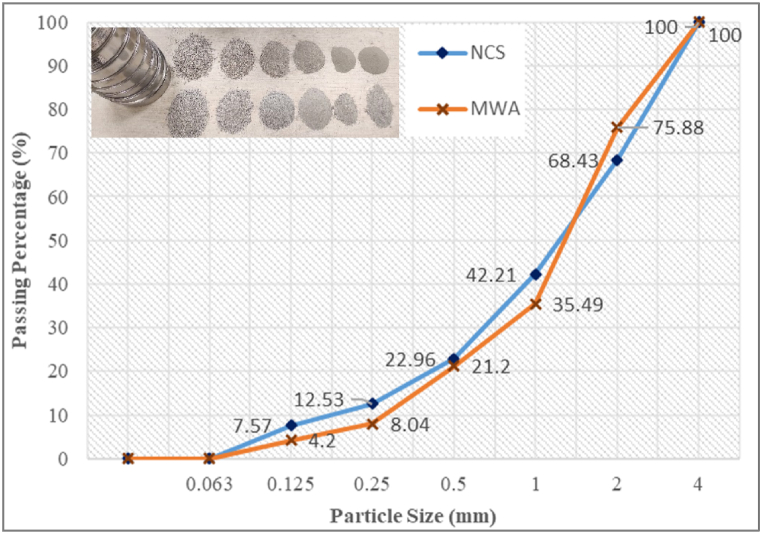
Sieve analysis of NCS and MWA.

CEM-I 42.5R cement, natural crushed sand (NCS) and MWA substituted in varying proportions to natural crushed sand were used in the self-compacting mortar (SCM) mixtures produced within the scope of the experimental study (see Fig. 1). The MWA used in the SCM design was first crushed into small pieces, then crushed in the crusher and sieved with a 4 mm sieve and a sieve analysis test was performed with the sieved material. The determined free weight of MWA and NCS were 2.50 and 2.58 g/cm^3^ respectively. The sieve analysis of NCS and MWA used for SCM production is given in Fig. 2 [44].

The chemical composition of the cement used in the mix design is given in Table 1. Tunceli city mains water in accordance with TS EN 1008 was used as mixing water in the study [45]. In the design of all SCM mixtures, the water/binder ratio (W/B) was taken as 0.44 and Sika Visco Crete Hi-Tech-28 polycarboxylate based high range water reducer (HRWR) conforming to TS EN 934-2+A1 was used to provide SCM properties [46].

**Table 1:** CEM-I 42.5R Cement properties.

Chemical Compounds (%)	SiO_2_	19.79
	Al_2_O_3_	3.16
	Fe_2_O_3_	4.15
	CaO	62.02
	Na_2_O	0.98
	SO_3_	2.98
	K_2_O	0.77
	MgO	2.22
	Cl^−^	0.01
Insoluble residue		0.60
Ignition lost		3.32
Blaine (cm^2^/g)		3804
Specific Gravity (g/cm^3^)		3.15

### 2.2 Method

#### 2.2.1 Mix design, manufacturing, coding, and curing conditions

Depending on the varying MWA substitution rate, 6 different SCM blends were produced within the scope of the experimental study, with one SCM blend being the reference. The mass substitution rate of MWA to SCM was designed to be 0, 5, 10, 20, 30, 40 % in increasing ratios. The W/B ratio in the SCM sets was taken as constant and 0.44. In addition, the amount of Portland cement used in all SCM sets was determined as 550 kg/m^3^. The viscosity and fresh state criteria of EFNARC [43] were achieved in this study by using variable proportions of HRWR since the W/B ratio was fixed. A laboratory type mixer with a capacity of 3 dm^3^ were used in the production of SCM sets. The specimens produced in all SCM sets designed by considering the SCM criteria of EFNARC standard are 40 × 40 × 160 mm. The 1 m^3^ mix design of 6 different SCM sets is given in Table 2.

**Table 2:** SCM mix designs (kg/m^3^).

Mix ID	Cement	Water	HRWR	NCS	MWA
SCM1-0	550	242	4.40	1500	0.00
SCM2-5	550	242	4.57	1425	75
SCM3-10	550	242	4.67	1350	150
SCM4-20	550	242	4.81	1200	300
SCM5-30	550	242	5.17	1050	450
SCM6-40	550	242	5.78	900	600

All specimens were produced in the laboratory and fresh state determinations were made by mini-collapse test and V-huni test with reference to EFNARC [43] criteria. All SCM sets were placed in molds without vibration and compression. After 24 h, the samples taken from the molds were coded according to the curing conditions and mix design. Table 3 shows the relevant coding.

**Table 3:** Group test designs.

TEST	GROUPS				
	A	B	C	D	E
Specific gravity	**Y**	**Y**	**Y**	**Y**	**Y**
Porosity	**Y**	**Y**	**Y**	**Y**	**Y**
Soptivity	**Y**	**Y**	**N**	**N**	**Y**
Flexural strength	**Y**	**Y**	**Y**	**Y**	**Y**
Compressive strength	**Y**	**Y**	**Y**	**Y**	**Y**
XRD	**N**	**N**	**N**	**Y**	**Y**
A. Group: 3-day water cure					
B. Group: 7^−^day water cure					
C. Group: 7-day water cure followed by a 21-day laboratory air cure.					
D. Group: On day 7, after water curing, curing for 120 h at 60 °C in an oven with air circulation.					
E. Group: 28-day water cure					

Y: Yes, N: No.

Maturation of the specimens was based on 5 different curing conditions. Groups A and B of the samples were matured with lime saturated water curing at 20 ± 2 °C for 3 and 7 days, respectively. Group C and D samples were removed from the tank after 7 days of lime saturated water curing, and Group C samples were kept in the laboratory until they were 28 days old. Group D specimens were matured by heat curing after 7 days of water curing. Group D samples were subjected to continuous 60 °C heat for 120 h in an air circulation oven and then kept in the laboratory environment until they reached constant temperature. Group E specimens were matured with lime-saturated water curing at 20 ± 2 °C for 28 days without interruption. All specimens matured under different curing conditions were subjected to a series of mechanical, physical and durability tests. In addition, XRD evaluation was performed for SCM1-0, SCM3-10, SCM4-20, SCM6-40 set (D and E Group).

#### 2.2.2 Experimental application

The fresh state workability properties of all SCM sets produced were evaluated by mini-slump flow test and V-funnel test according to EFNARC criteria [43]. In addition, hardened state properties were determined by unit volume weight, porosity, capillary water absorption, flexural tensile strength, and compressive strength tests. Evaluations for all test groups were performed for different curing and specimen ages.

##### 2.2.2.1 Fresh SCM experiments

A series of experimental studies were carried out to determine the conformity of the fresh state properties to EFNARC [43] for 6 different SCMs produced. In SCMs, the V-funnel provides fresh state determination based on the flow time (s) of the mortar through the funnel hopper, while the mini slump spread test provides fresh state determination based on the slump spread diameter of the fresh mix. The range of funnel flow time (7–11 s) and mini-slump spread diameter range (240–260 mm) recommended by EFNARC [43] criteria for SCM design were achieved for all SCM mixes by keeping the HRWR ratio variable and the W/B ratio constant as 0.44.

##### 2.2.2.2 Hardened SCM experiments

Physical, mechanical and durability properties were determined with 40 × 40 × 160 mm prism specimens of all SCM specimen sets. After curing, the oven dry, saturated dry surface and weight in water of the specimens were determined and oven dry unit volume weight, porosity, capillary water absorption, flexural tensile [47] and then compressive strength [48] tests were performed. Unit volume weight, porosity and water absorption tests were performed on specimens of all SCM sets matured after 3 days water curing (A group), 7 days water curing (B group) and 28 days water curing (E group). In addition, flexural tensile and compressive strenght tests were performed for groups A, B, C, D, E after all curing conditions of all SCM sets. Experiments were carried out with 3 specimens in each group of each SCM set and the data were converted into equations and graphs.

Capillary water absorption tests were carried out with 3 samples each for groups A, B and E of the SCM sets matured at 20 ± 2 °C in water for 3, 7 and 28 days, respectively. For the capillary water absorption test, the water-cured specimens were dried in an air-circulating oven at 110 ± 2 °C for 24 h after determining their suspended weight in water and air (dry water saturated surface). After drying, the samples were removed from the oven and allowed to cool in a controlled manner in the laboratory environment and the oven dry weights were determined. Three specimens for each set of SCM were insulated on four sides with a waterproof layer. Capillary water absorption was achieved by contacting one of the parallel and uninsulated surfaces with water. According to ASTM C1585-13 [49], capillary water absorption data of samples taken from a single surface in contact with water were determined at specific time intervals (5, 10, 30, 60, 240 and 1440 min) with an accuracy of 0.01 g. Graphs related to water absorption, water absorption surface area and time relationship were obtained for groups A, B, E of all SCM sets.

The samples of SCM1-0, SCM3-10 SCM4-20 and SCM6-40 microstructure analysis of analyzed by X-ray Diffractometer (XRD) with RIGAKU-Miniflex 600 X-ray diffractometer at Munzur University MUNTEAM. The specimens used in the analyses were obtained by bringing the specimens used in the compressive strength test to sufficient size and quantity after the test.

## 3 Results and discussion

### 3.1 Results of fresh state specifications

For all SCM sets, the V-funnel flow time (s) and mini-slump-spreading cone spreading diameter (mm) data, which are determinants of fresh state properties, are as given in Table 4. As in similar studies [9,50,51], the high-water absorption requirement of MWA increased the water requirement in the mix design. The resulting reduced workability reduces the control of the effective W/B ratio of the mortar matrix. This was achieved using HRWR in the SCMs in this study. The V-funnel flow time (7–11 s) and mini-slump spread diameter (240–260 mm) were maintained in all SCM sets. The SCM set with the longest V-funnel flow time is the reference set. Also, since the SCM6-40 set contains 40 % substituted MWA, the mini-spread diameter is at the reference limit. Increasing the MWA content in the blend shortened the V-funnel flow time and decreased the mini-spreading diameter value. The increased use of HRWR due to the increased amount of MWA has an impact on these results. MWA has more surface roughness and finer particles as it is obtained by crushing processes [52]. As in similar study results, MWA [[53], [54], [55], [56]] and plasticizer usage [54,55,57] influence viscosity.

**Table 4:** Fresh state test results.

Mix ID	V-funnel time (s)	Slump flow diameter (mm)
SCM1-0	10.49	258
SCM2-5	9.8	256
SCM3-10	9.31	252
SCM4-20	8.7	248
SCM5-30	8.2	244
SCM6-40	7.8	238

### 3.2 Porosity test results

**Fig. 3:**
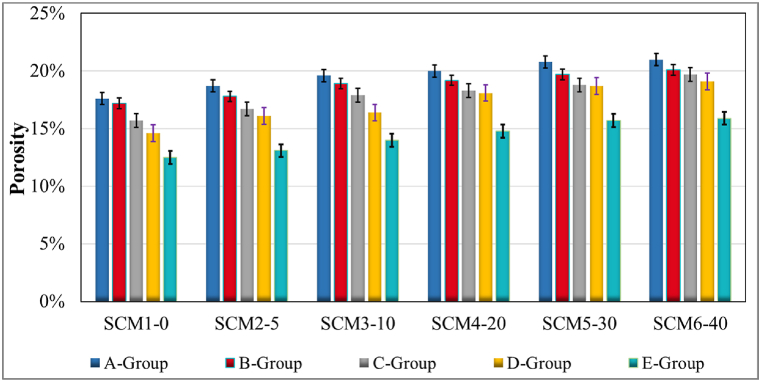
Porosity test results.

The porosity data determined for all groups of all SCM sets are as given in Fig. 3. Porosity data were obtained from 40 × 40 × 160 mm prism specimens of all sets and groups. According to the data obtained; the porosity increases in MWA substituted sets is linear compared to the reference set SCM1-0. The least porosity data was obtained from group E samples of SCM1-0 set. MWA increase is effective on porosity increase. In addition, according to the curing conditions, the porosity rate in the groups with 28 days water curing is the lowest for all sets. Prolonged water curing decreases porosity. In addition, in SCM4-20 and SCM5-30 sets with 20 % and 30 % MWA substitution, the effect of heat curing and keeping in laboratory environment after 7 days of water curing has a similar effect on porosity. Except these two sets, the porosity ratio of group C specimens is higher than the porosity ratio of group D specimens in other sets. However, this excess is in the range of 0.015-0.006. As in similar studies, the porosity ratio increases with increasing MWA [58]. Heat curing influences the physical properties of mortar specimens such as porosity [59]. The porosity ratio of the heat cured Group D specimens was lower than the porosity ratio of Group C specimens kept in the laboratory. This effect due to heat curing was obtained in all sets.

The increase in porosity with increasing MWA is due to the need for MWA to absorb more water than aggregate. The water that does not react and surrounds the MWA like a film layer causes the porosity to increase. This is generally associated with the irregular and coarse surface of recycled aggregate [50,60].

### 3.3 Unit volume weight results

**Fig. 4:**
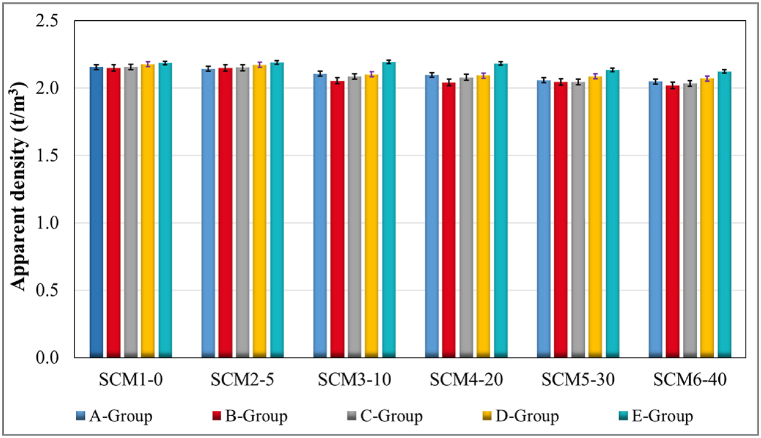
Unit weight results.

Dry unit volume weight data for all SCM sets and all groups are given in Fig. 4. According to the data obtained; the rate of increase in MWA substitution is associated with a decrease in unit weight data. The highest unit volume weight value (2.195 tons/m^3^) was obtained in Group E specimens of SCM3-10 set, which was water cured for 28 days. In Group A specimens, the unit volume weight value decreased from 2156 to 2048 with a decreasing trend from the SCM1-0 set to the SCM6-40 set. This change was approximately 5.0 %, while for Group B, C, D and E samples it was 6 %, 5.7 %, 5 %, and 3 % respectively. This can be explained by the substitution of MWA with NCS by mass and the 3 % difference between the free weights of MWA and NCS. In addition, Group E specimens subjected to water curing for 28 days gave the highest unit volume weight data in all sets. The water/cement ratio is influenced by the absorption of possibly higher amounts of water by MWAs. This affects both the cement hydration process and consequently the final mechanical and physical properties [[61], [62], [63], [64]]. Free water, which does not react chemically, moves away from the non-water cured specimen over time. This has an effect on the unit volume weight and porosity. This is the reason why the unit volume weight is highest in group E specimens subjected to lime-saturated water curing.

Accordingly, there is an increase in the SCM3-10 set with 10 % MWA substitution compared to the reference set SCM1-0. In all sets, water curing up to 28 days has a positive effect on the unit weight value. These results are similar to the effect of substituted products on unit weight in aggregate. In addition, the water absorbed by MWA, which is not chemically reacted, may have caused a decrease in unit volume weight over time, especially in laboratory (C group) and heat-treated (D group) samples. As in similar studies, the water removed from the samples influences the unit volume weight [65,66]. Also, the change in unit volume weight could be due to the difference in the unit volume weight of MWA substituted with NCS.

### 3.4 Tensile strength results in flexure

**Fig. 5:**
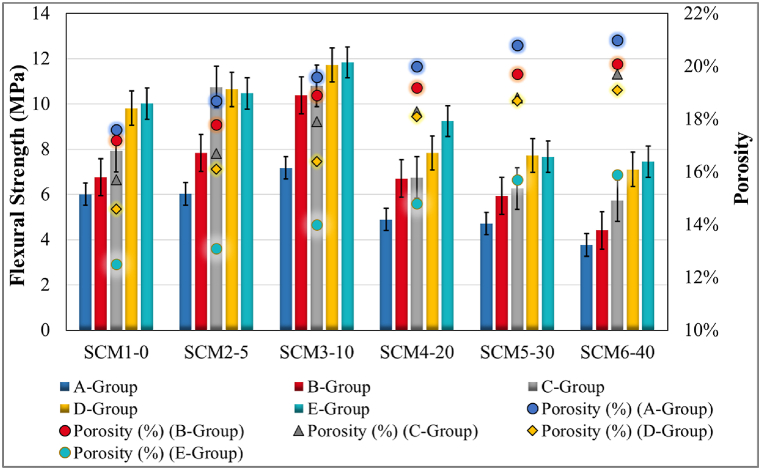
Flexure strength vs porosity results.

The relationship between porosity and tensile strength in flexure prepared with the experimental data of SCM sets for all specimen ages and curing conditions is given in Fig. 5. For specimens cured for 3 days (Group A), 7 days (Group B) and 28 days (Group E) in lime-saturated standard water, the increase in flexural tensile strength with increasing water curing time is valid for all SCM sets. In addition, the flexural tensile strength of specimens group C is lower than the flexural tensile strength of specimens Group E or specimens Group D. Only the SCM2-5 set is an exception for this case. In general, porosity decreases with prolonged water curing and increases with increasing MWA substitution. The decrease in flexural tensile strength is significant during and after 20 % MWA substitution. This reduction in porosity has a positive effect on flexural tensile strength. Flexural tensile strength increases from A to B and from B to E with prolonged water curing in all sets. The data on flexural tensile strength are similar to the existing studies in the literature [[67], [68], [69], [70]].

### 3.5 Compressive strength test results

**Fig. 6:**
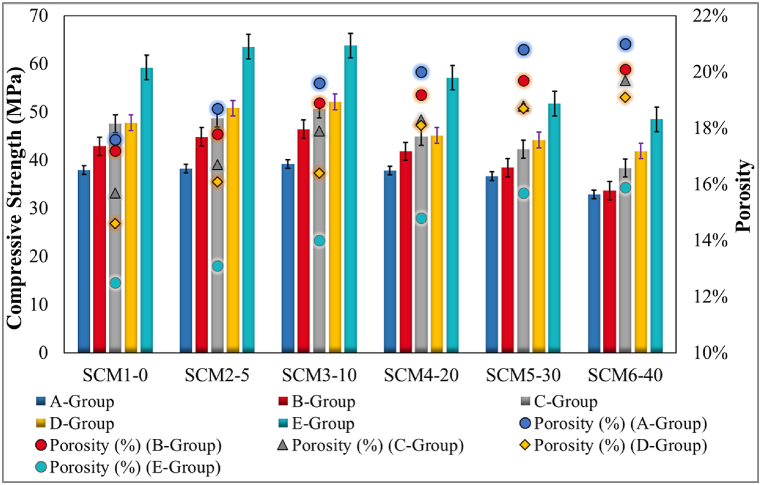
Compressive strength vs porosity results.

The effect of replacement ratios on compressive strength test data and porosity is as shown in Fig. 6. Compared to the reference set, SCM2-5 and SCM3-10 sets present higher compressive strength test data values for all curing conditions. SCM2-5 and SCM3-10 sets contain 5 % and 10 % MWA substitution respectively. Group E specimens of the SCM3-10 set provide the highest compressive strength test data with 63.83 MPa. The prolonged lime-saturated water cure influences porosity and the compressive strength increases with decreasing porosity in all MWA sets. The compressive strength of MWA increases up to 10 % substitution. For other substitution conditions, the ultimate compressive strength after 28 days is lower than the reference set. The use of MWA influences the mechanical performance and the results agree with similar studies in the literature [51,71,72].

### 3.6 Sorptivity test results

**Fig. 7:**
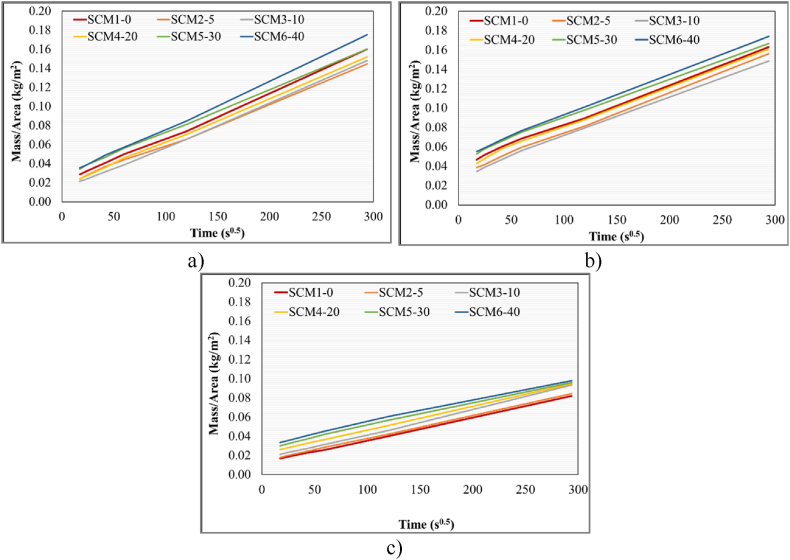
**a.** 3-Day (A group), **b.** 7-Day (B group), **c.** 28-Day (E group), sorptivity test results.

Capillary water absorption tests were performed with specimens cured for 3 days (Group A), 7 days (Group B) and 28 days (Group E). Fig. 7a. Capillary water absorption of group A specimens in relation to time (s^0.5^) and mass/area. At the end of 24 h, the SCM set with the highest capillary water absorption rate from a single 40 × 40 mm surface was SCM6-40 with 40 % MWA substitution. The lowest water absorption rate belongs to the SCM2-5 set. Capillary water absorption rate is related to the porosity and therefore the amount of MWA in the mortar mix. Increasing the amount of MWA increases the water demand, which in turn affects the capillary water absorption potential [73]. The capillary water absorption test data of Group B specimens after 7 days of water curing are given in Fig. 7b. The highest amount of capillary water absorption in Group B specimens belongs to SCM6-40 set at the end of 24 h as in Group A specimens. The lowest amount of water absorption in Group B specimens was recorded in the SCM3-10 set at the end of 24 h. The data obtained at the end of the capillary water absorption test performed with E group specimens are as shown in Fig. 7c. As in Group A and B specimens, the highest amount of water absorption after 24 h of capillary water absorption test was obtained in the SCM6-40 set in Group E specimens. Among the specimens subjected to 3-, 7- and 28-days water curing, the lowest water absorption belongs to group C specimens as shown in Fig. 7c. Prolonged water curing has a decreasing effect on the amount of capillary water absorption. This is related to the decreasing porosity ratio between group A, B and E specimens. In addition, increasing MWA substitution has a more pronounced effect on capillary water absorption especially after 30 % (SCM5-30) and 40 % (SCM6-40) substitution rate. SCM5-30 and SCM6-40 sets have the highest amount of water absorption in group A, B and E specimens.

To determine the sorption coefficient the mass gain divided by the surface area of the top surface is plotted versus the exposure time. The sorption coefficients are defined as shown in the following Eq (1) and Eq (2):

(1) $$I=S\sqrt{t}+b\;\left(\text{point}\;\text{measured}\;\text{during}\;\text{the}\;\text{first}\;\text{day}\;\text{are}\;\text{used},\text{excluding}\;\text{the}\;\text{point}\;\text{at}\;\text{origin}\right)$$

(2) $$\frac{W}{\rho A}=S\sqrt{t}+I_{0}$$

W is gives as mass gain (kg), A is gives as surface are tested (m^2^), t is gives as time variable (min), S is gives as sorption coefficient $\left(\frac{\text{mm}}{\sqrt{\min}}\right)$, I_0_ is gives as initial sorption (mm), and ρ is gives as density of water (kg/m^3^)

**Fig. 8:**
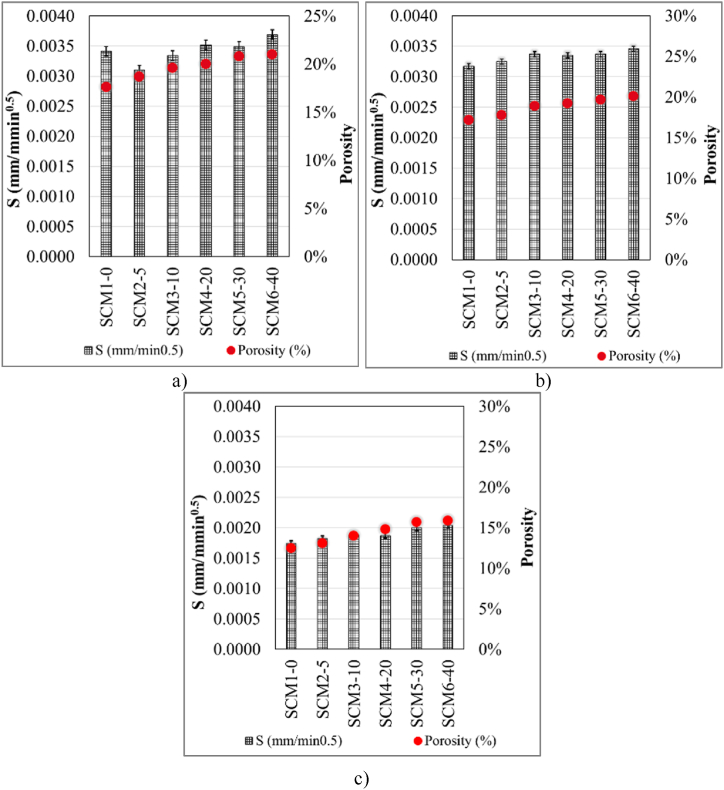
**a.** 3-Day (A group), **b.** 7-Day (B group), **c.**28-Day (E group), I_0_ and porosity values.

Porosity values and S coefficient data of Group A specimens of 6 different SCM mixtures after 3 days of water curing are given in Fig. 8a. According to Fig. 8a.; MWA substitution causes an increase in porosity. In addition, the S value is highest in the SCM6-40 set and lowest in the SCM2-5 set. The S value for group A specimens of all sets is between 0.00341 and 0.00369. The data obtained contain similar results with the studies in the literature. The high-water absorption requirement of MWA may lead to shrinkage with advancing sample age and [74]consequently to an increase in the amount of water absorption [42,66]. Porosity and S values of Group B specimens of all SCM mixtures after 7 days of water curing are given in Fig. 8b. As can be seen in Fig. 8b, porosity increases with increasing MWA ratio, and the lowest porosity ratio belongs to the reference set. In addition, the S value also increases from the SCM1-0 series to the SCM6-40 set. The S value for group B specimens of all sets is between 0.00317 and 0.00346. Porosity and S data of 28-day water cured group E specimens from 6 different SCM mixtures are given in Fig. 8c. There is an increase in porosity and S value with increasing MWA ratio. With the increase in MWA, the water demand and porosity of SCMs increase [58]. The increased water demand is usually improved by using plasticizers. However, as in similar studies [10,[12], [13], [14],[16], [17], [18],[75], [76], [77], [78], [79]], increasing HRWR influences porosity in this study.

The S value of all SCM sets is between 0.00174 and 0.00204. When group A, B and E samples of SCM sets are correlated; prolonged water cure leads to a decrease in S value and porosity. This can be explained by the formation of Calcium-silica-hydrate (CSH) gels with prolonged water cure.

**Fig. 9:**
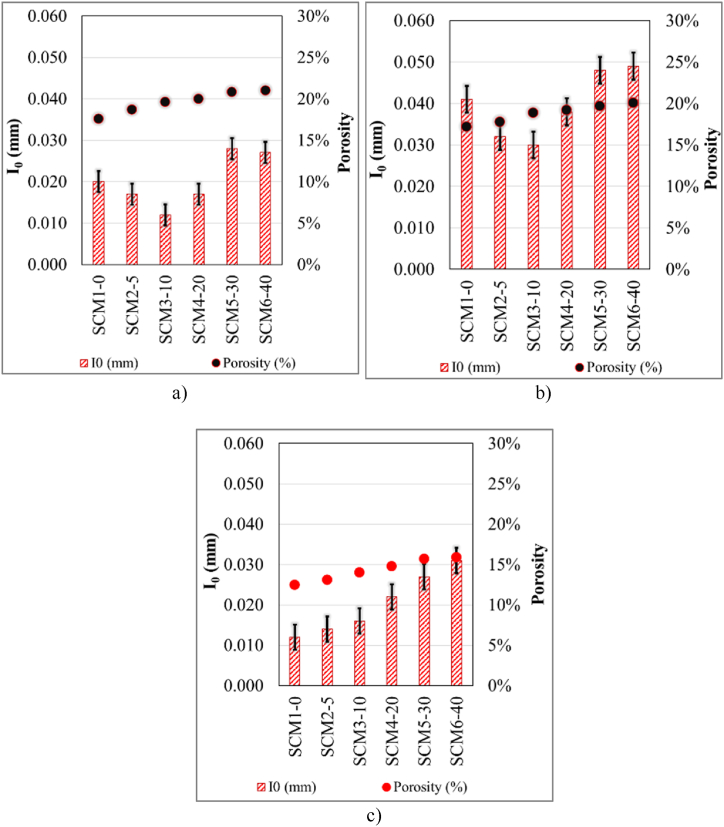
**a.** 3-Day (A group), **b.** 7-Day (B group), **c.**28-Day (E group), I_0_ and porosity values.

The correlation graphs of porosity and I_0_ data of SCM sets after 3, 7 and 28 days of water curing are given in Fig. 9. The porosity of group A specimens increased with increasing MWA. The highest value of porosity was recorded in SCM6-40 while the highest value of I_0_ was recorded in SCM5-30. Also, the lowest I_0_ value (0.012) belongs to SCM3-10 set (Fig. 9a). The highest porosity value in group B samples of SCM sets subjected to 7 days water curing belongs to SCM6-40 set where MWA is the highest (Fig. 9b). The decreasing I0 value among SCM1-0, SCM2-5 and SCM3-10 sets follows an increasing trend towards SCM4-20, SCM5-30, and SCM6-40. The data obtained from group E specimens for which porosity and I_0_ values were determined after 28 days water curing of all SCM sets are given in Fig. 9c. The increase in porosity with increasing MWA exhibits a similar trend as in group A and B specimens. However, the porosity ratio decreased in all SCM sets with prolonged water curing time. The fluctuations in the I_0_ value of group A and B specimens exhibit an increasing linearity in group E specimens. Porosity and I_0_ value increase with increasing MWA. The lowest porosity and I_0_ values belong to the reference set (SCM1-0). After 28 days of water curing, the lowest and highest porosity ratios are 0.125 and 0.159, respectively. In addition, the I_0_ value takes the lowest and highest value for SCM1-0 and SCM6-40 set, respectively. These values are 0.12 and 0.31 respectively.

### 3.7 XRD test results

**Fig. 10:**
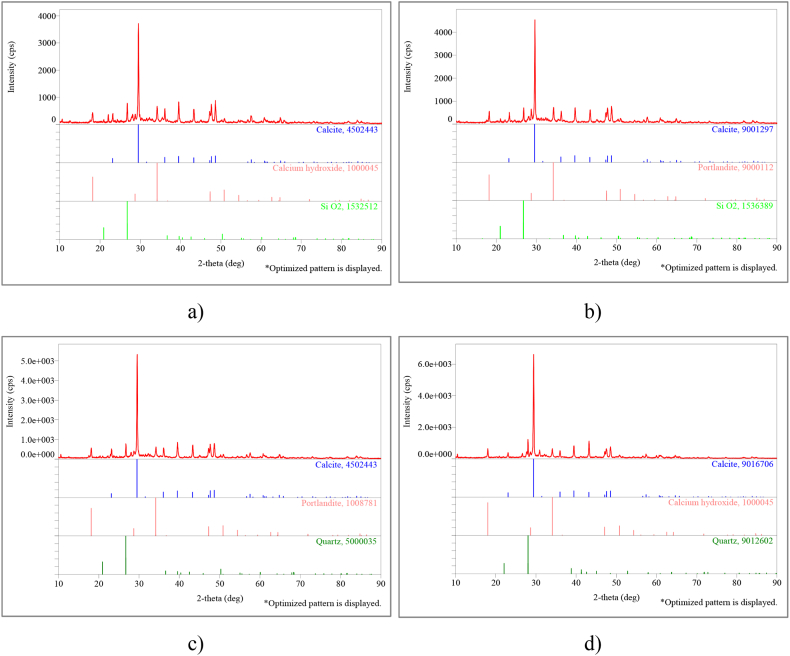
XRD results (E group) for **a.** SCM1-0, **b.** SCM3-10, **c.** SCM4-20, **d.** SCM6-40 sets.

**Fig. 11:**
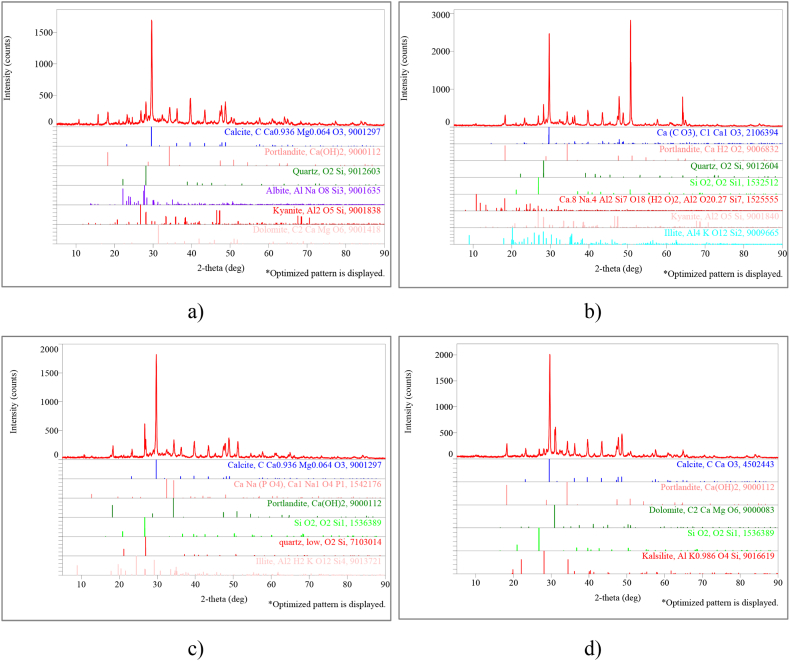
XRD results (D group) for a. SCM1-0, b. SCM3-10, c. SCM4-20, d. SCM6-40 sets.

The data of the SCM sets whose mineral composition was determined by X-ray diffraction are shown in Fig. 10, Fig. 11. XRD evaluation of the sets with 28-day water cure (E group) and heat cure (D group) was performed for the SCM1-0, SCM3-10, SCM4-20, SCM6-40 sets (Fig. 10, Fig. 11). As can be seen from the XRD results of the samples taken from the mixes after water cure, the main compounds are quartz (SiO_2_), calcite (CaCO_3_) and calcium hydroxide Ca(OH)_2_ and Portlandite (Fig. 10). Portlandite is the naturally occurring form of calcium hydroxide (Ca(OH)_2_). These phases were found to have the most intense peaks occurring at 2θ = 26.75° (quartz), 2θ = 29.40° (calcite) and 2θ = 30.80° (dolomite).

XRD results obtained from the SCM sets (group D) subjected to 7-day water curing followed by temperature curing show that the main compounds are similarly quartz, calcite and calcium hydroxide and Portlandite (Fig. 11). Although much less frequently observed, illite has been recorded as a mineralogical structure with a protective effect on porosity and permeability and occurs as an occurrence in cement structure [[80], [81], [82]]. Albite, like pyrite and jarrosite, appears to have a generally ambiguous affinity with carbonate; gibbsite has the same concentration in most samples [83]. Other studies in the literature [83] show that albite, like pyrite and jarrosite, has a generally uncertain affinity with carbonate. Based on the Figure-11, this is similar to the situation detected in the XRD analysis of the samples and can be found in samples taken as components from natural rocks.

## 4 Conclusion

In the 6 different SCM sets produced within the scope of the experimental study, MWA substituted into the aggregate after mechanical disintegration and grinding was used. A series of experimental studies were carried out at different ages to determine the fresh state and hardened state. In addition, microanalytical evaluations were carried out with hardened specimens. According to the results of the experimental studies.

• An increase in porosity was observed in all sets where MWA was substituted compared to the reference set (SCM1-0). Continuation of the water curing period until the 28th day decreases the porosity rate. The porosity ratio of the heat cured (D group) and laboratory air cured (C group) groups are in close range.
• Although the oven dry unit weight is generally lower than the reference set for groups A, B, C, D, the values are close to the reference set for the samples after 28 days of water curing (group E). This is evident in the sets with 30 % and 40 % MWA substitution where the oven dry unit volume weight is lower than the reference set for all curing conditions.
• The extended water curing time improves the mechanical performance for all MWA substitution conditions. Tensile strength in flexure increases with increasing water curing time for all substitution rates. Lime saturated water curing improves the mechanical performance in all sets. In the 28-day specimens of all groups (groups C, D and E), the flexural tensile strength is highest in group E, except for 5 % substitution.
• Although the capillary water absorption rate shows an increasing trend with increasing MWA, the lowest capillary water absorption rate falls in mixtures with 10 % substitution.
• Although the capillary water absorption rate shows an increasing trend with increasing MWA, the lowest capillary water absorption rate decreases up to blends with 10 % substitution and then increases.

## CRediT authorship contribution statement

**Melek Akgül:** Writing – review & editing, Supervision, Resources, Methodology, Funding acquisition.

## Declaration of competing interest

The authors declare that they have no known competing financial interests or personal relationships that could have appeared to influence the work reported in this paper.
